# The prognostic value of ITGA and ITGB superfamily members in patients with high grade serous ovarian cancer

**DOI:** 10.1186/s12935-020-01344-2

**Published:** 2020-06-18

**Authors:** Tingting Zhu, Ruifang Chen, Jieyu Wang, Huiran Yue, Xin Lu, Jun Li

**Affiliations:** 1 Department of Gynecology, Obstetrics and Gynecology Hospital of Fudan University, No. 419, Fangxie Road, Shanghai, 200011 China; 2 Shanghai Key Laboratory of Female Reproductive Endocrine Related Diseases, Shanghai, 200011 China

**Keywords:** High grade serous ovarian cancer, Integrin, ITGA, ITGB, Prognosis, Nomogram

## Abstract

**Background:**

Deregulation of integrins signaling had been documented to participate in multiple fundamental biological processes, and the aberrant expression of integrin family members were linked to the prognosis of various cancers. However, the role of integrins in predicting progression and prognosis of ovarian cancer patients are still largely elusive. This study is aimed to explore the prognostic values of ITGA and ITGB superfamily members in high grade serous ovarian cancers (HGSOC).

**Methods:**

GSE26712 dataset was used to determine the differential expression of ITGA and ITGB superfamily member between HGSOC and normal counterparts. The Cancer Genome Altas (TGGA) and GSE9891 datasets were used to determine the prognostic values of ITGA and ITGB superfamily members in HGSOC, followed by the development of nomograms predictive of recurrence free survival (RFS) and overall survival (OS).

**Results:**

ITGA6 and ITGB5 expression were significantly downregulated in HGSOC compared with that in normal counterparts. In contrast, ITGA2, ITGA5, ITGA7, ITGA8, ITGA9, ITGA10, ITGB3, ITGB4, ITGB6, and ITGB8 were all significantly upregulated in HGSOC compared with that in normal counterparts. Both univariable and multivariable analysis indicated that ITGB1 was associated with extended RFS. The ITGB1-related nomogram indicated that ITGB1 had the largest contribution to RFS, followed by FIGO stage and debulking status. The C-index for predicting RFS was 0.55 (95% CI 0.50–0.59) in TCGA dataset (training dataset) and 0.65 (95% CI 0.59–0.72) in GSE9891 dataset (validation dataset), respectively. Regarding OS, ITGB8 was associated with reduced survival suggested by both univariable and multivariable analysis. ITGA7 appeared to be associated with improved survival though without reaching statistical significance. The ITGA7/ITGB8-based nomogram showed that age at initial diagnosis had the largest contribution to OS, followed by ITGB8 and ITGA7 expression. The C-index for predicting OS was 0.65 (95% CI 0.60–0.69) in TCGA dataset (training dataset) and 0.59 (95% CI 0.51–0.66) in GSE9891 dataset (validation dataset), respectively.

**Conclusion:**

In conclusion, ITGB1, ITGA7 and ITGB8 added prognostic value to the traditional clinical risk factors used to assess the clinical outcomes of HGSOC.

## Background

High grade serous ovarian cancer (HGSOC) is an aggressive and incurable malignancy and most patients with newly diagnosed HGSOC presented with advanced stage [1]. The mainstay of primary treatment is debulking surgery with the aim of complete resection, followed by platinum-based chemotherapy [2]. Despite initial chemosensitivity, only about 30% of the patients were still alive 5 years after initial diagnosis. The prognosis of the patients were closely correlated with the intrinsic and acquired molecular characteristics of ovarian cancer tissues [1, 3]. Especially in the era of precision medicine, an improved understanding of the molecular features of ovarian cancer had led to better stratification of the patient prognosis and subsequent identification of novel therapeutic targets [4]. For example, breast related cancer antigens (BRCA) mutation and homologous recombination deficiency (HRD) status had been exploited to develop poly-adenosine diphosphate (ADP) ribose polymerase (PARP) inhibitors to treat HGSOC patients individually, leading to significant improvement in survival [5–7]. However, some patients with HGSOC still had a poor prognosis despite of novel treatments [8]. It is important to identify new biomarkers to predict HGSOC prognosis, which will subsequently facilitate the development of new personalized treatment strategies [4].

Integrins are heterodimeric transmembrane receptors composed of an alpha subunit and a beta subunit. They are involved in cell adhesion and signaling. Deregulation of integrins signaling had been documented to participate in various processes of cancer, including but not limited to tumor initiation, metastatic cascade, and drug resistance [9]. Given their critical roles in multiple fundamental biological processes, the aberrant expression of integrin family members were linked to the prognosis of various cancers [10–12]. However, the role of integrins in predicting progression and prognosis of HGSOC patients are still largely elusive [13].

In this study, we assessed the prognostic values of ITGA and ITGB, two superfamily of integrins, in HGSOC by resorting to the high-throughput expression data deposited in Gene Expression Omnibus(GEO) database and The Cancer Genome Altas (TCGA) database.

## Materials and methods

### Datasets used in present study

GSE26172 dataset, GSE9891 dataset, and TCGA dataset were obtained from the “curatedOvarianData” Bioconductor package (version 2.12 for R 3.0.3). GSE26712 dataset was used to determine the differential expression of ITGA and ITGB superfamily members between HGSOC (n = 185) and normal human ovarian surface epithelium (HOSE) (n = 10). The prognostic value of ITGA and ITGB superfamily members were evaluated and validated in TCGA dataset (n = 405) and GSE9891 dataset (n = 135), respectively. The prognostic values of ITGB1 and ITGB8 were further validated using Kaplan–Meier plotter database [14]. The expression of ITGB1 was detected using the mean value of five probes, including 215878_at, 215879_at, 216178_x_at, 211945_s_at and 216190_x_at in Kaplan–Meier plotter database. The expression of ITGB8 was detected using the mean value of two probes, including 205816_at and 211488_s_at in Kaplan–Meier plotter database.

### Statistical analysis

Detailed methods of statistical analysis were described in our previous published paper [15]. All the statistical analyses were performed using IBM SPSS Statistics (version 22.0) and R (version 3.5.2). All factors with P values < 0.15 in the univariable analysis were entered into the multivariable Cox regression analysis. Prognostic factors with P values < 0.10 indicated by multivariable analysis, and two established prognostic factors, including FIGO stage and debulking status, were incorporated to develop the prognostic nomogram. P values < 0.05 are considered statistically significant. All reported P values are two-sided.

## Results

### The differential expression of ITGA and ITGB superfamily members between HGSOC and HOSE

**Fig. 1:**
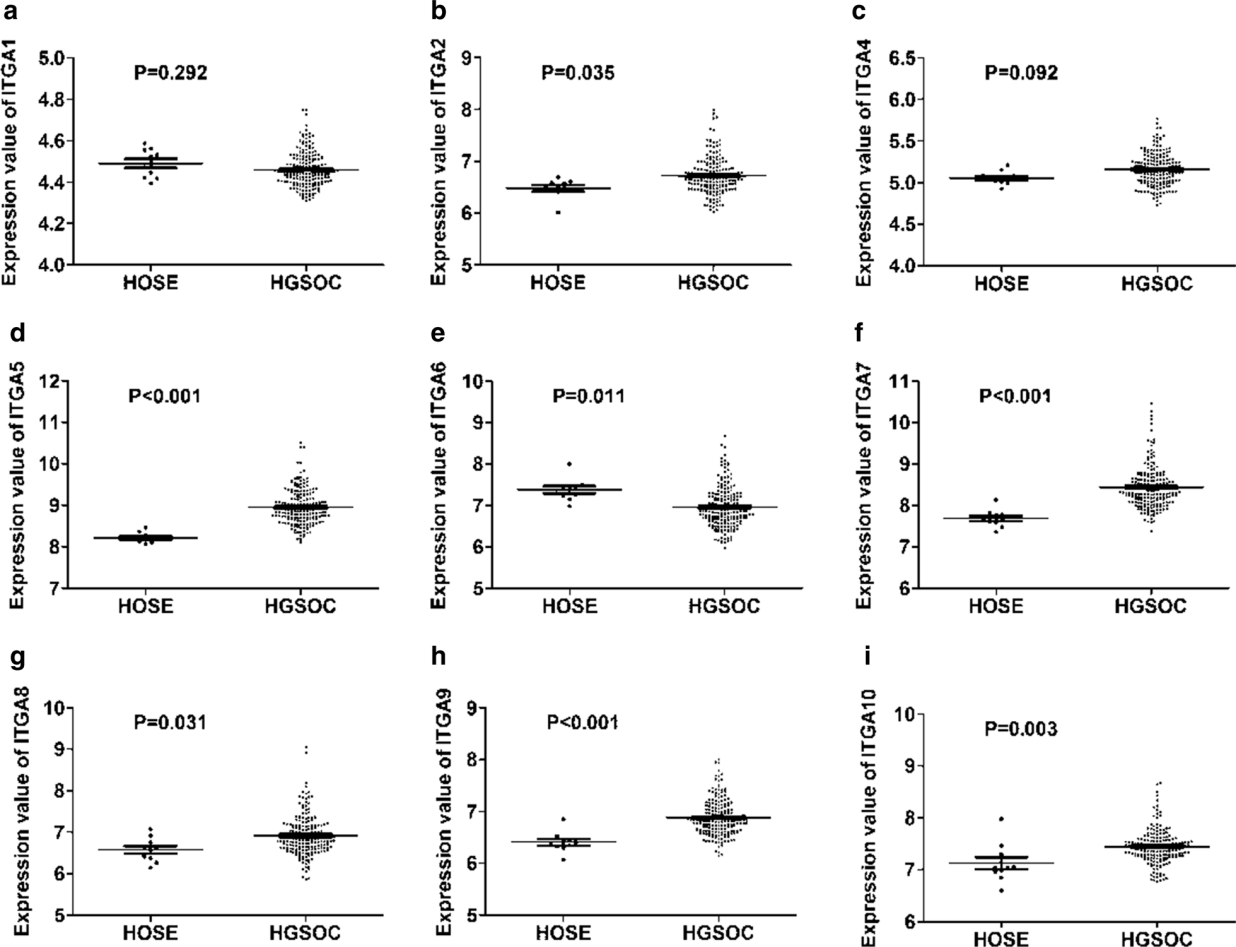
Differential expression of ITGA superfamily members between HGSOC and normal counterparts. (**a**–**i**) There was no significant difference in the expression of ITGA1 (**a**) and ITGA4 (**c**) between HGSOC and normal counterparts. ITGA2 (**b**), ITGA5 (**d**), ITGA7 (**f**), ITGA8 (**g**), ITGA9 (**h**), and ITGA10 (**i**) expression were significantly increased in HGSOC compared with that in normal counterparts. In contrast, ITGA6 (**e**) was significantly decreased in HGSOC compared with that in normal counterparts

**Fig. 2:**
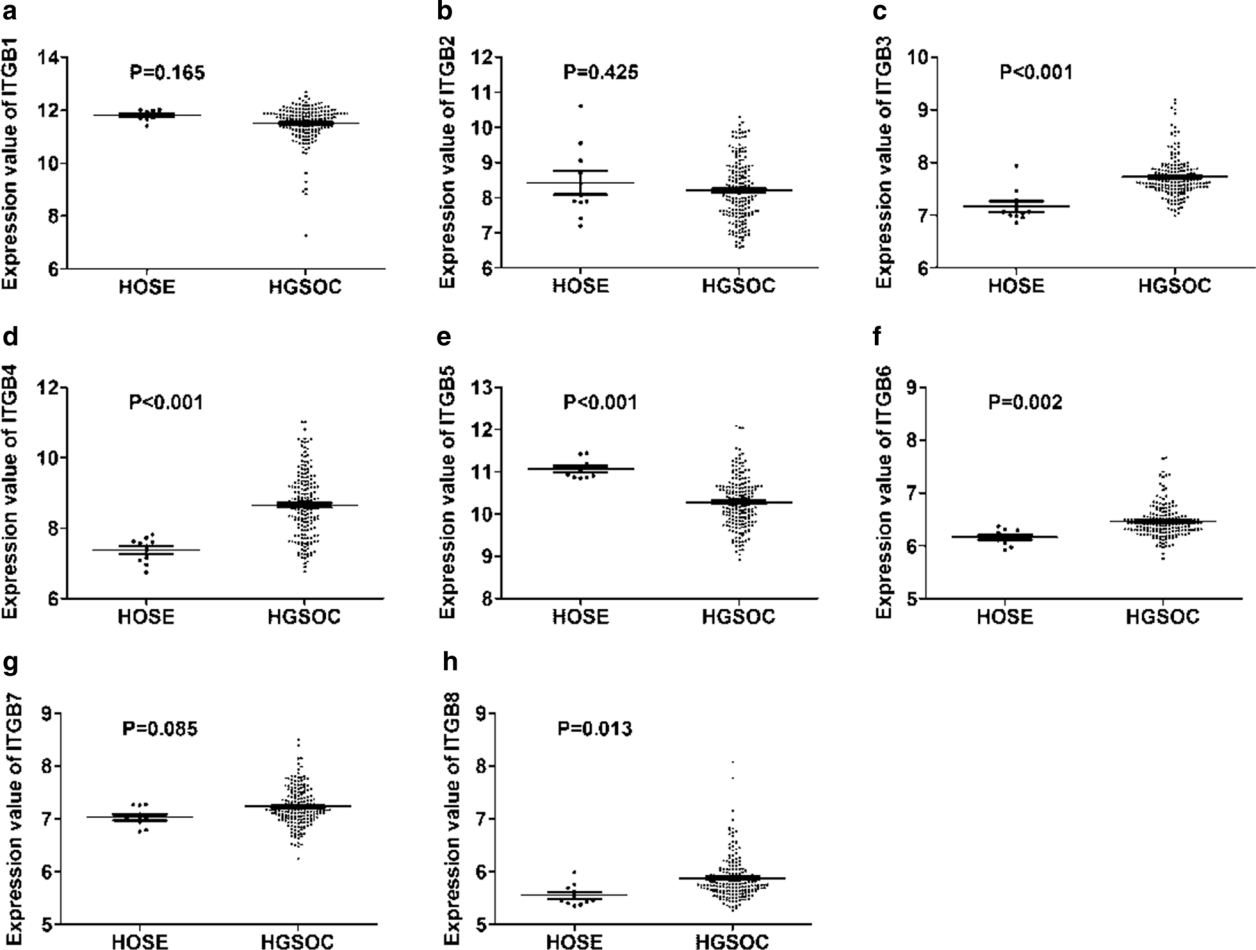
Differential Expression of ITGB superfamily members between HGSOC and normal counterparts. **a**–**h** There was no significant difference in the expression of ITGB1 (**a**), ITGB2 (**b**), and ITGB7 (**g**) between HGSOC and normal counterparts. ITGB3 (**c**), ITGB4 (**d**), ITGB6 (**f**), and ITGB8 (**g**) were significantly overexpressed in HGSOC compared with that in normal counterparts. In contrast, ITGB5 (**e**) was significantly downregulated in HGSOC compared with that in normal counterparts

We first determined the differential expression of ITGA and ITGB superfamily members between HGSOC and HOSE using GSE26712 dataset. There was no significant difference in the expression of ITGA1 (Fig. 1a), ITGA4 (Fig. 1c), ITGB1 (Fig. 2a), ITGB2 (Fig. 2b), and ITGB7 (Fig. 2g) between SOCs and HOSE. ITGA2 (Fig. 1b), ITGA5 (Fig. 1d), ITGA7 (Fig. 1f), ITGA8 (Fig. 1g), ITGA9 (Fig. 1h), ITGA10 (Fig. 1i), ITGB3 (Fig. 2c), ITGB4 (Fig. 2d), ITGB6 (Fig. 2f), and ITGB8 (Fig. 2g) expression were significantly increased in HGSOC compared with that in HOSE. In contrast, ITGA6 (Fig. 1e) and ITGB5 (Fig. 2e) were significantly decreased in HGSOC compared with that in HOSE.

### The prognostic value of ITGA and ITGB members for recurrence free survival (RFS) in HGSOC

Next, we determined the prognostic significance of ITGA and ITGB members in predicting RFS in patients with HGSOC using TCGA dataset (Table 1). In univariable analysis, advanced FIGO stage and increased ITGB1 expression were associated with decreased RFS. In multivariable analysis, increased ITGB1 expression were associated with decreased RFS. Advanced FIGO stage tended to be related to decreased RFS though without reaching statistical significance.

**Table 1:** The prognostic significance of ITGA and ITGB superfamily members in predicting RFS in HGSOC patients in TCGA dataset

Variables	Number of patients	Univariate analysis		Multivariate analysis	
		HR (95% CI)	P value	HR (95% CI)	P value
Age	405	1.005 (0.993–1.018)	0.391	/	/
Stage			0.035		0.065
Early	23	1		1	
Late	382	2.255 (1.060–4.796)		2.046 (0.956–4.379)	
Debulking			0.546		/
Optimal	302	1		/	/
Suboptimal	103	1.100 (0.807–1.501)		/	/
ITGA1	405	0.882 (0.570–1.362)	0.570	/	/
ITGA2	405	0.778 (0.528–1.147)	0.205	/	/
ITGA4	405	1.060 (0.679–1.655)	0.798	/	/
ITGA5	405	1.152(0.950–1.396)	0.150	/	/
ITGA6	405	0.863 (0.723–1.029)	0.100	0.888 (0.739–1.068)	0.207
ITGA7	405	1.086 (0.916–1.287)	0.341	/	/
ITGA8	405	1.040 (0.714–1.515)	0.838	/	/
ITGA9	405	1.054 (0.754–1.451)	0.788	/	/
ITGA10	405	0.893 (0.618–1.292)	0.549	/	/
ITGB1	405	1.327 (1.002–1.757)	0.048	1.341 (1.018–1.767)	0.037
ITGB2	405	1.039 (0.928–1.162)	0.506	/	/
ITGB3	405	0.769 (0.451–1.311)	0.335	/	/
ITGB4	405	1.054 (0.936–1.186)	0.385	/	/
ITGB5	405	1.080 (0.911–1.280)	0.375	/	/
ITGB6	405	0.874 (0.659–1.159)	0.349	/	/
ITGB7	405	0.834 (0.614–1.133)	0.245	/	/
ITGB8	405	1.124 (0.962–1.314)	0.142	0.888 (0.739–1.068)	0.207

### Generation and validation of ITGB1-related nomogram predictive of RFS

**Fig. 3:**
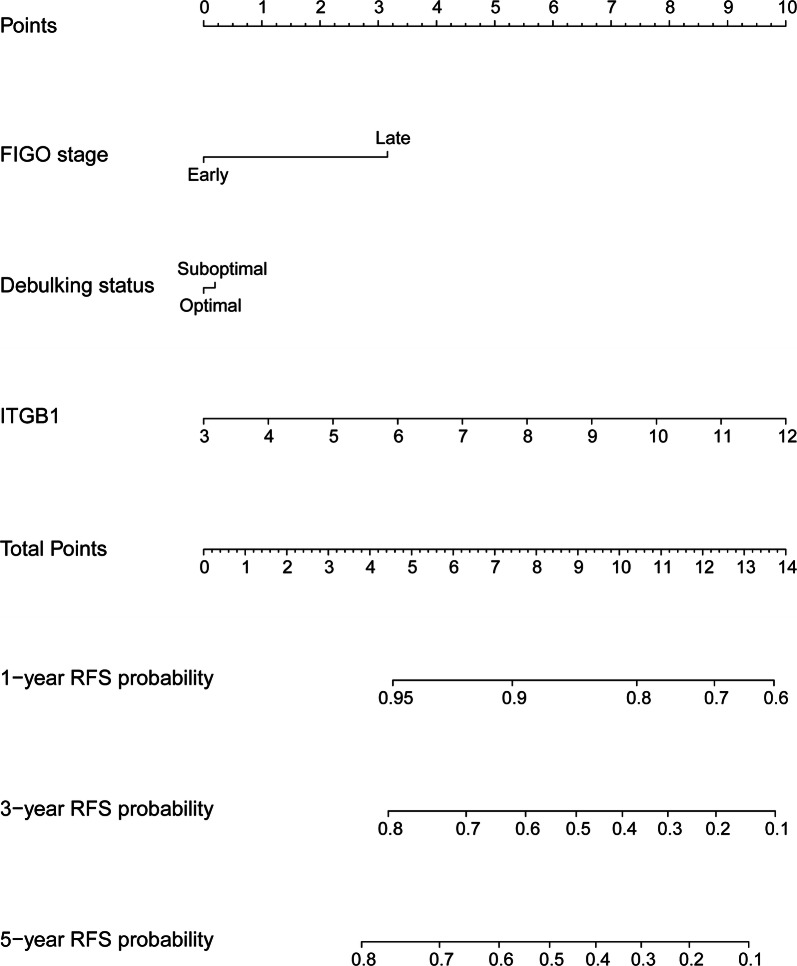
Generation of ITGB1-related nomogram predictive of RFS. The nomogram illustrated that ITGB1 had the largest contribution to RFS, followed by FIGO stage and debulking status

To quantitatively predict the prognosis of HGSOC, a nomogram was generated and validated in the training dataset (TCGA) and validation dataset (GSE9891) respectively. The predictors included FIGO stage, debulking status, and ITGB1 expression. Among these, ITGB1 had the largest contribution to RFS, followed by FIGO stage and debulking status (Fig. 3). The C-index for predicting RFS was 0.55 (95% CI 0.50–0.59) in TCGA dataset (training dataset) and 0.65 (95% CI 0.59–0.72) in GSE9891 dataset (validation dataset), respectively.

### The prognostic value of ITGA and ITGB members for overall survival (OS) in HGSOC

Next, we assessed the prognostic value of ITGA and ITGB members in predicting OS in patients with HGSOC using TCGA dataset (Table 2). In univariable analysis, elder age, suboptimal debulking, and ITGB8 overexpression were associated with decreased OS. Advanced FIGO stage appeared to be associated with a worse outcome though without reaching statistical significance. In multivariable analysis, elder age and ITGB8 overexpression remained to be independent predictors of decreased OS.

**Table 2:** The prognostic significance of ITGA and ITGB superfamily members in predicting OS in HGSOC patients in TCGA dataset

Variables	Number of patients	Univariate analysis		Multivariate analysis	
		HR (95% CI)	P value	HR (95% CI)	P value
Age	405	1.025 (1.013–1.038)	< 0.001	1.023 (1.010–1.036)	< 0.001
Stage			0.078		0.116
Early	23	1		1	
Late	382	2.079 (0.922–4.685)		1.934 (0.850–4.403)	
Debulking			0.017		0.230
Optimal	302	1		1	
Suboptimal	103	1.430 (1.065–1.920)		1.209 (0.887–1.649)	
ITGA1	405	0.745 (0.474–1.170)	0.202	/	/
ITGA2	405	1.037 (0.713–1.508)	0.851	/	/
ITGA4	405	1.174 (0.777–1.774)	0.446	/	/
ITGA5	405	0.922 (0.759–1.120)	0.416	/	/
ITGA6	405	0.976 (0.819–1.163)	0.782	/	/
ITGA7	405	0.867 (0.729–1.033)	0.110	0.849 (0.712–1.013)	0.069
ITGA8	405	0.987 (0.682–1.429)	0.947	/	/
ITGA9	405	0.944 (0.660–1.348)	0.750	/	/
ITGA10	405	1.107 (0.756–1.622)	0.601	/	/
ITGB1	405	1.054 (0.832–1.336)	0.662	/	/
ITGB2	405	1.004 (0.901–1.118)	0.945	/	/
ITGB3	405	0.850 (0.501–1.441)	0.546	/	/
ITGB4	405	0.992 (0.881–1.117)	0.889	/	/
ITGB5	405	1.087 (0.916–1.290)	0.341	/	/
ITGB6	405	0.975 (0.759–1.253)	0.844	/	/
ITGB7	405	0.821 (0.592–1.139)	0.238	/	/
ITGB8	405	1.207 (1.045–1.394)	0.011	1.175 (1.014–1.362)	0.032

### Generation and validation of ITGA7/ITGB8-related nomogram predictive of OS

**Fig. 4:**
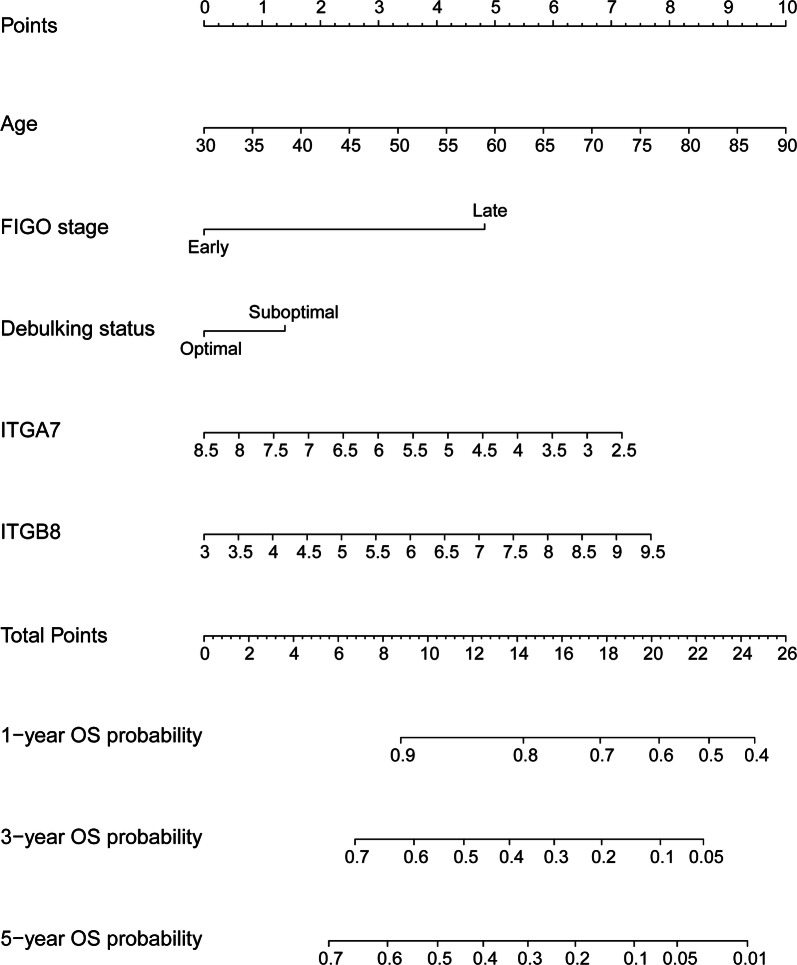
Generation of ITGA7/ITGB8-related nomogram predictive of OS. The nomogram illustrated that age at initial diagnosis had the largest contribution to OS, followed by ITGB8 and ITGA7 expression

To quantitatively predict the prognosis of HGSOC, a nomogram was generated and validated in the training dataset (TCGA) and validation dataset (GSE9891) respectively. The predictors included age at initial diagnosis, FIGO stage, debulking status, ITGA7 and ITGB8 expression. Among these, age at initial diagnosis had the largest contribution to OS, followed by ITGB8 and ITGA7 expression (Fig. 4). The C-index for predicting OS was 0.65 (95% CI 0.60–0.69) in TCGA dataset (training dataset) and 0.59 (95% CI 0.51–0.66) in GSE9891 dataset (validation dataset), respectively.

### Validation of the prognostic value of ITGB1 and ITGB8 using KM Plotter tool

**Fig. 5:**
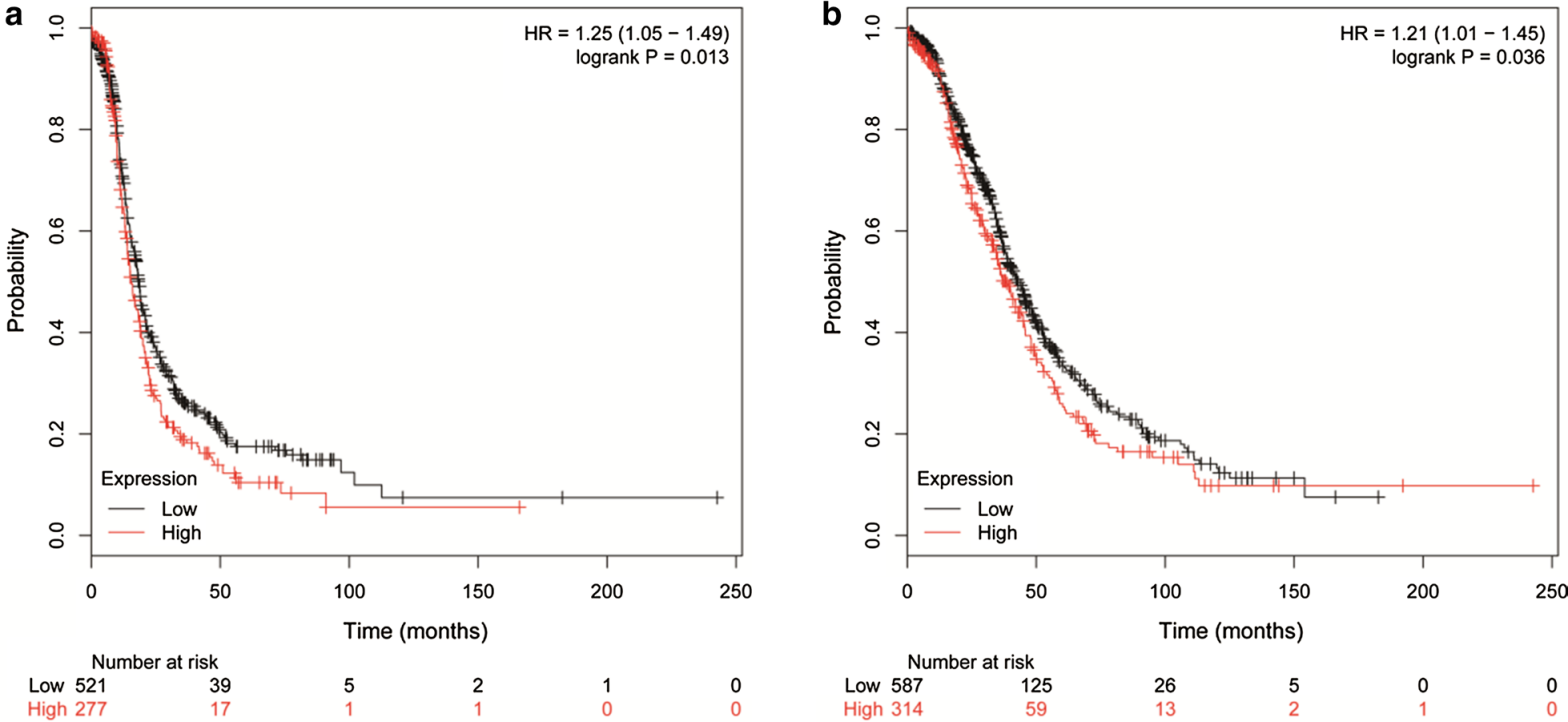
Validation of the prognostic value of ITGB1 and ITGB8 using KM Plotter tool. **a** Increased expression of ITGB1 was associated with decreased PFS; **b** Elevated level of ITGB8 was associated with decreased OS

Next, we validated the prognostic value of ITGB1 and ITGB8 using Kaplan–Meier plotter tool. Consistently, increased expression of ITGB1 was associated with decreased progression free survival (PFS) (Fig. 5a; HR, 1.25; 95% CI 1.05–1.49), and elevated level of ITGB8 was associated with decreased OS (Fig. 5b; HR, 1.21; 95% CI 1.01–1.45).

## Discussion

The present study indicated that increased ITGA7 expression appeared to be associated with improved OS by multivariable analysis though without reaching statistical significance. Both univariable and multivariable analysis indicated that ITGB1 and ITGB8 was an independent predictor of RFS and OS in HGSOC, respectively. For RFS, the ITGB1-related nomogram indicated that ITGB1 had the largest contribution to RFS, followed by FIGO stage and debulking status. For OS, the ITGA7/ITGB8-based nomogram showed that age at initial diagnosis had the largest contribution to OS, followed by followed by ITGB8 and ITGA7 expression. These two nomograms could quantitatively predict the prognosis of HGSOC.

Indeed, the associations between some integrin family members and the clinical outcomes of ovarian cancer patients had been assessed previously. However, these studies usually evaluated the prognostic value of a single integrin member in a relatively small sample size. In contrast, our study assessed and validated the prognostic value of ITGA and ITGB, two superfamily of integrins, in serous ovarian cancer patients in a relatively large sample size by resorting to the high throughput microarray data in both TCGA and GEO datasets. This may yield more reliable results and generalize these findings with reduced doubts.

Since their discovery in the late 1980s, the integrin signaling have been demonstrated to be involved in multiple cellular processes of cancer development and progression [9]. In ovarian cancer, Yang et al. showed that ITGB1 expression was upregulated in ovarian cancer, and overexpression of ITGB1 enhanced the invasion of ovarian cancer cells [16]. Moreover, downregulation of ITGB1 impaired tumor growth and peritoneal spread in in vivo assays [16]. Increased expression of ITGB1 was associated with drug resistance in ovarian cancer cells [17]. This may partly explain the correlation between reduced RFS and ITGB1 overexpression in ovarian cancer patients revealed by our data. Similar findings were observed in other types of cancers [18, 19]. In ovarian cancer, ITGB8 had been demonstrated to be associated with drug resistance. Overexpression of ITGB8 restored cisplatin resistance inhibited by miR-199a-3p in SKOV-3 cells [20]. This may partly explain the association between reduced OS and ITGB8 overexpression in ovarian cancer patients [21]. Indeed, similar findings had been observed in other types of cancers. For example, ectopic expression of ITGB8 enhanced proliferation and invasion of colorectal cancer cells [22]. Consistently, ITGB8 was also upregulated in glioblastoma tissues and its upregulation was associated with worse clinical outcome [23]. ITGB8 inhibition impaired self-renewal ability, stemness, migration, and tumor formation capacity [23]. Collectively, the above findings indicated that ITGB8 functioned as oncogenes in various cancers and may become a potential therapeutic target in cancer treatment.

FIGO stage is a key determinant for prognostic prediction in HGSOC. Even though patients with the same FIGO stage were managed similarly, their clinical outcomes varied greatly. These differences in prognosis might be attributed to the biological heterogeneity of ovarian cancer. In this study, we found that ITGB1 expression had larger contributions to RFS than FIGO stage. Combination of ITGB1, FIGO stage and debulking status could predict RFS more accurately. Similarly, combination of clinical risk factors and ITGB8 expression may also predict OS more accurately.

## Conclusions

In conclusion, ITGB1, ITGA7 and ITGB8 added prognostic value to the traditional clinical risk factors used to assess the clinical outcomes of HGSOC. Future works are needed to explore the functions of ITGB1, ITGA7 and ITGB8 in HGSOC.

## Data Availability

All the data are deposited in TCGA and GEO databases.
